# Electrocortical measures of win and loss processing are associated with mesocorticolimbic functional connectivity: A combined ERP and rs‐fMRI study

**DOI:** 10.1111/psyp.14118

**Published:** 2022-06-07

**Authors:** Natania A. Crane, Katie L. Burkhouse, Stephanie M. Gorka, Heide Klumpp, K. Luan Phan

**Affiliations:** ^1^ Department of Psychiatry University of Illinois at Chicago Chicago Illinois USA; ^2^ Department of Psychiatry and Behavioral Health The Ohio State University Columbus Ohio USA

**Keywords:** anxiety, depression, ERP, functional connectivity, resting‐state, reward

## Abstract

The reward positivity (RewP) event‐related potential is a well‐validated measure of reward processing implicated in internalizing psychopathologies. The RewP is thought to reflect reward reactivity in the mesocorticolimbic system; however, it is not clear how the RewP is related to the functional connectivity of reward‐related brain regions. The current study examined associations between the RewP (Win and Loss residuals) and resting‐state fMRI (rs‐fMRI), among adults with internalizing psychopathology (IP) and healthy controls (HC). All participants (*N* = 102) completed a validated monetary reward task during electroencephalogram and rs‐fMRI. Regression analyses were conducted with (1) RewP‐Win residual amplitude and striatal seeds (caudate, putamen, nucleus accumbens) and (2) RewP‐Loss residual amplitude and anterior cingulate cortex (ACC) seeds. Overall, individuals with greater RewP‐Win residual amplitude demonstrated increased rs‐fMRI connectivity between striatal regions and the medial prefrontal cortex, as well as the parahippocampal gyrus, but decreased connectivity between striatal regions and regions involved in cognitive control and sensorimotor processing. Greater RewP‐Loss residual was related to greater connectivity between the ACC and regions involved in reward/loss processing and motor control, but decreased connectivity between the ACC and regions involved in cognitive control. Relationships between the RewP and rs‐fMRI were generally consistent across IP and HC. However, a few patterns were unique to IP. Results indicate the RewP is associated with resting‐state functional connectivity of reward‐ and loss‐related brain regions, suggesting connectivity of the mesocorticolimbic system may be an important individual difference factor in dimensions of attainment of reward and loss.

## Abstract 2

Our results provide the first preliminary evidence that the RewP, a neurophysiological marker of initial response to reward and loss, is associated with functional connectivity of the mesocorticolimbic system during resting‐state fMRI among individuals with and without internalizing psychopathologies. Mesocorticolimbic connectivity may be an important individual difference factor in determining how individuals respond to the attainment of reward and loss.

## 1 INTRODUCTION

Reward dysfunction is implicated across the spectrum of anxiety and depression disorders (e.g., Craske et al., 2016). To better understand reward‐related processes, RDoC has identified several biologically based reward constructs within the Positive Valence System, including initial responsiveness to reward attainment. One measure that is frequently used to examine initial reward responsiveness at the neurophysiological level is the reward positivity (RewP). The RewP is an event‐related potential (ERP) component maximal at frontocentral electrode sites occurring approximately 250–350 ms following the receipt of a reward. The RewP is thought to reflect the processing of positive feedback (e.g., monetary reward) versus breaking even or losing (see Proudfit, 2015). Accumulating evidence suggests that the RewP has excellent psychometric properties (Bress, Meyer, & Proudfit, 2015) and is a valid measure of individual differences in reward processing. For example, higher RewP values have been correlated with positive emotionality in children (Kujawa et al., 2015) and self‐reported reward sensitivity in college students (Bress & Hajcak, 2013). The RewP has been shown to be disrupted (e.g., blunted) in internalizing psychopathologies, especially in depression in clinical and non‐clinical samples (Bress et al., 2013; Bress, Meyer, & Hajcak, 2015; Bress, Meyer, & Proudfit, 2015; Burkhouse et al., 2017; Foti & Hajcak, 2009; Nelson et al., 2016). The RewP has also been identified as a biological marker of depression risk among youth (for review, see Kujawa & Burkhouse, 2017) and adults (for review, see Proudfit, 2015). Few studies have examined the RewP in relation to anxiety, especially among clinical samples. Among studies examining the relationship between the RewP and anxiety, the findings are mixed, with one study showing an attenuated RewP is associated with greater trait anxiety among young adults (Gu et al., 2010), other studies finding that a larger RewP is associated with higher symptoms of social anxiety among children and undergraduate students (Kessel et al., 2015; Nelson & Jarcho, 2021), and other studies finding no relationship between the RewP and anxiety symptoms among children, adolescents, or undergraduate students (Bress et al., 2012; Bress, Meyer, & Hajcak, 2015; Foti & Hajcak, 2009). In a clinical sample of adult participants with a variety of internalizing diagnoses, we found that an attenuated RewP was associated with greater affective distress/misery‐based symptoms, but not fear‐based anxiety symptoms (Burkhouse et al., 2017). Taken together, there is mixed evidence as to whether the RewP is associated with anxiety. Few studies have examined whether the RewP is disrupted among individuals with comorbid depression and anxiety, despite the fact that comorbidity of these disorders is extremely common (Kalin, 2020).

The RewP was previously termed the feedback negativity (FN), as traditionally the RewP was analyzed as the difference in average reactivity to rewards versus losses, while the FN was the inverse: The difference in average reactivity to losses versus rewards (see Proudfit, 2015). Recent psychometric studies suggest that reactivity to rewards and losses can be better analyzed using a regression‐based approach (Meyer et al., 2017). Specifically, this approach computes two sets of residuals reflecting (a) neural reactivity to reward independent of reactivity to loss (RewP‐Win residual), and (b) neural reactivity to loss independent of reactivity to reward (RewP‐Loss residual). This approach is better able to isolate neural activity related to a specific process of interest than the difference scores because the Win and Loss residuals obtained from this regression‐based approach are orthogonal. Importantly, the residual scores also have superior psychometric properties compared to traditional difference scores (Bress, Meyer, & Proudfit, 2015; Ethridge & Weinberg, 2018).

Source localization studies suggest that the RewP (Win > Loss) is related to reward‐related brain activity. For example, one study found that the RewP may originate from the dorsal striatum (Foti et al., 2011), which includes the caudate and putamen. Follow‐up studies found the RewP was related to fMRI BOLD and EEG activation (via source localization) of brain regions implicated in rewards, such as the ventral striatum (nucleus accumbens [NAcc]) and medial prefrontal cortex (mPFC), during reward attainment among healthy individuals (Becker et al., 2014; Carlson et al., 2011; Foti et al., 2011; Gehring & Willoughby, 2002). Together, these findings provide compelling evidence that the RewP may be generated by neural activity in reward‐related regions, especially the striatum. On the other hand, source localization studies suggest that the FN (Loss > Win) may originate from the anterior cingulate cortex (ACC) (Dehaene et al., 1994; Miltner et al., 1997), a region involved in error processing and cognitive control. Notably, these previous source localization and combined electroencephalogram (EEG)‐fMRI BOLD studies did not examine if the RewP is related to individual differences in functional connectivity between brain regions. Although these studies have identified brain reward regions involved in reward and loss processing, it is not clear how the connections of these brain regions influence reward and loss processing. The intrinsic connectivity of brain regions related to reward and loss responsivity can help us to better understand the network of brain regions involved in reward and loss processing. Examining intrinsic connectivity has advantages over examining task‐based neural activation (using EEG or fMRI), as it is reliable and can be more easily compared across studies and diagnostic groups (i.e., does not use a task that differs across studies and/or diagnostic groups). Indeed, growing evidence suggests neural intrinsic connectivity, as measured by resting‐state fMRI (rs‐fMRI), is an important tool for understanding distinct and common neurobiological profiles across psychiatric diagnoses (Peterson et al., 2014), which can help in informing prevention and intervention targets. Understanding the relationship between rs‐fMRI and the RewP can increase precision of the meaning of the RewP. Furthermore, most previous studies examining neural correlates of the RewP have focused exclusively on psychiatrically‐healthy individuals. Examining the relationship between intrinsic brain reward connectivity (i.e., resting‐state) and neural activity during initial responsiveness to reward and loss attainment in individuals with a range of anxiety or depression symptoms and healthy individuals is an important step toward a better understanding of individual differences in neural circuitry associated with reward and loss processing.

Therefore, the current study examined associations between the RewP (Win and Loss residuals) and rs‐fMRI connectivity in reward and loss‐related brain regions among a community sample of adults with and without internalizing psychopathologies. Specifically, we examined the relationship between RewP‐Win residual and rs‐fMRI connectivity using striatal seeds (based on a prior study showing the RewP [Win >Loss] may originate from the striatum; Foti et al., 2011) to all other frontolimbic regions, and we examined the relationship between RewP‐Loss residual and rs‐fMRI connectivity using ACC seeds (based on prior studies that the FN [Loss >Win] may originate from the ACC; Dehaene et al., 1994; Miltner et al., 1997) to all other frontolimbic regions. We hypothesized that individuals with a larger RewP‐Win residual during reward would exhibit greater resting‐state functional connectivity between the striatum and mPFC (reward‐related brain regions). We also hypothesized the individuals with a larger RewP‐Loss residual would exhibit greater resting‐state functional connectivity between the ACC and other regions functionally connected to the ACC and involved in loss processing (i.e., striatum, anterior insula, mPFC, and inferior frontal gyrus; Dugre et al., 2018). Furthermore, we explored whether these relationships differed by diagnostic group (i.e., healthy controls (HC) versus individuals with internalizing psychopathology (IP)), though we did not expect differences in associations based on diagnostic group given the dimensional nature of the RewP based on theory and previous studies.

## 2 METHOD

### 2.1 Participants

All participants in this study were free from psychotropic medication and major medical and neurological illness as confirmed by a Board‐Certified physician. As part of a larger study that was funded by, and designed to be consistent with, the NIMH RDoC initiative, IP were enrolled if they met full threshold or sub‐threshold criteria (all but one criterion for a specific disorder) for at least one depressive or anxiety disorder, reported a total score of ≥23 on the Depression, Anxiety, and Stress Scale (DASS‐21; Lovibond & Lovibond, 1995) had a Global Assessment of Functioning score of ≤60, and were seeking treatment. Exclusion criteria for HC included a current or lifetime Axis I disorder. Exclusion criteria for all participants were less than 18 or more than 65 years of age, contraindications to magnetic resonance imaging (e.g., pregnancy, ferrous objects), current substance use disorder (within 6 months of the study), history of other major psychiatric illness (e.g., bipolar disorder, psychotic disorders), or current cognitive dysfunction (e.g., traumatic brain injury, pervasive developmental disorder). The University of Illinois at Chicago Institutional Review Board approved the study, and informed consent was obtained from all participants. All participants were compensated for their time and all procedures complied with the Helsinki Declaration.

One hundred and twenty‐four participants were enrolled in the study and completed both fMRI and EEG measures. Ten participants were excluded for excessive motion during the resting‐state fMRI scan, defined as >2 mm displacement in any direction. Twelve additional participants were excluded due to poor quality EEG data, defined as having fewer than 15 artifact‐free trials per condition. The final sample comprised 102 participants: 71 IP and 31 HC (see Table 1). Almost all IP met criteria for a disorder. Five IPs met subthreshold criteria for their primary disorder (three for Generalized Anxiety Disorder and two for Panic Disorder [PD])‐ among these only one IP with subthreshold PD did not meet full criteria for another disorder. All measures were collected within 2 weeks of the fMRI scan and before receiving pharmacotherapy or psychotherapy as part of the larger study. Participants were required to have a negative urine drug screen at the time of screening and prior to EEG and fMRI.

**TABLE 1:** Participant characteristics

	Adult participants	IP	HC	*p*‐Value
	(*N* = 102)	(*n* = 71)	(*n* = 31)	
Age	26.17 (8.75)	27.24 (8.83)	23.71 (8.17)	.06
Years of education	15.96 (2.96)	16.15 (3.11)	15.52 (2.61)	.32
Gender (% female)	63%	67%	52%	.12
Ethnicity (% Hispanic)	18%	17%	19%	.77
Race				.07
% Caucasian	50%	56%	35%	–
% More than 1 race or unknown	6%	5%	10%	–
% African‐American	23%	24%	20%	–
% Asian	17%	11%	32%	–
% American Indian/Alaskan Native	4%	4%	3%	–
Clinical diagnoses				
No diagnosis	33%	–	100%	–
MDD (principal, present)	14%, 41%	14%, 41%	–	–
Dysthymia (principal, present)	2%, 5%	2%, 5%	–	–
GAD (principal, present)	28%, 49%	28%, 49%	–	–
PTSD (principal, present)	5%, 13%	5%, 13%	–	–
SAD (principal, present)	16%, 27%	16%, 27%	–	–
PD (principal, present)	2%, 12%	2%, 12%	–	–
Reward task variables (mean amplitude)				
Win	16.97 (8.34)	16.78 (8.68)	17.41 (7.63)	.73
Loss	12.94 (7.33)	13.14 (7.94)	12.50 (5.77)	.69
ΔRewP (Win‐Loss)	4.03 (5.62)	3.65 (6.18)	4.91 (3.98)	.30
RewP‐Win residual	−0.42 (5.52)	−0.78 (5.99)	0.40 (4.20)	.32
RewP‐Loss residual	0.00 (4.84)	−0.73 (2.95)	0.32 (5.46)	.21

*Note*: All values are means and standard deviations unless otherwise noted.

Abbreviations: GAD, generalized anxiety disorder; HC, healthy controls; IP, individuals with internalizing psychopathology; MDD, major depressive disorder; PD, panic disorder; PTSD, posttraumatic stress disorder; RewP, reward positivity; SAD, social anxiety disorder.

### 2.2 Assessment of psychopathology

Lifetime diagnoses of Axis I disorders were assessed via the Structured Clinical Interview for DSM‐5 Disorders (SCID‐5; First et al., 2015) by a master's‐level clinician or PhD/MD assessor. Consistent with the RDoC initiative (Kozak & Cuthbert, 2016), comorbidity was permitted (see Table 1). Symptoms of depression and anxiety were assessed with the 17‐item Hamilton Depression Rating Scale (HAM‐D; Hamilton, 1960) and the 14‐item Hamilton Anxiety Rating Scale (HAM‐A; Hamilton, 1959), respectively. Not surprisingly, individuals with a clinical diagnosis (IP) had more depression (*M* = 12.23, *SD* = 4.30; mild severity) than HCs (*M* = 0.39, *SD* = 0.67) (*F*[1101] = 231.24, *p* < .001) and anxiety (*M* = 17.55, *SD* = 6.87; mild to moderate severity) than HCs (*M* = 0.94, *SD* = 1.53), *F*(1,101) = 176.66, *p* < .001. However, IP and HC did not differ in demographic characteristics including age, years of education, gender, and race/ethnicity (see Table 1).

### 2.3 EEG reward task

Participants completed a validated reward‐guessing game, the “Doors” task (see Proudfit, 2015), during EEG that consisted of 40 trials. On each trial, participants were asked to choose one of two doors shown side by side on a computer monitor; the graphic remained visible until a choice was made. A fixation mark then appeared for 1000 ms, followed by a feedback screen for 2000 ms. Feedback consisted of either a green “↑”, indicating a win of $.50, or a red “↓”, indicating a loss of $.25; these amounts were chosen to give gains and losses equivalent subjective values (Tverksy & Kahneman, 1992). After receiving feedback, a fixation mark was presented for 1500 ms, followed by a screen reading “Click for the next round,” which remained on‐screen until participants responded. Participants received 20 trials each of gain and loss feedback, presented in a random order.

### 2.4 EEG data acquisition and preprocessing

**FIGURE 1:**
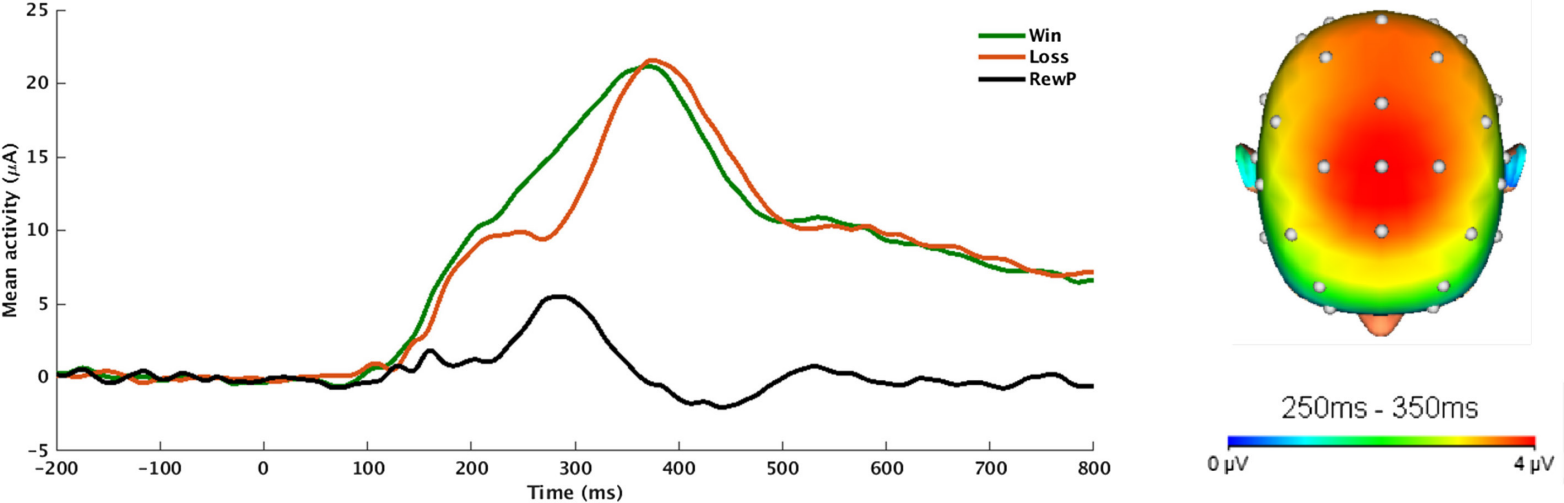
Response‐locked ERP waveform for win and loss trials, as well as the difference wave (reward positivity; ΔRewP) across the entire sample (*n* = 102) on the left. Topographic scalp maps of neural activity depict the win minus loss difference across the entire sample.

Continuous EEG was recorded during the task using an elastic cap and the ActiveTwo BioSemi system (BioSemi, Amsterdam, Netherlands). Thirty‐four standard electrode sites were used. One electrode was placed on each mastoid. The EEG signal was pre‐amplified at the electrode to improve the signal‐to‐noise ratio. The data were digitized at 24‐bit resolution with a Least Significant Bit (LSB) value of 31.25 nV and a sampling rate of 1024 Hz, using a low‐pass fifth‐order sinc filter with a −3 dB cutoff point at 204.8 Hz. Off‐line analyses were performed using Brain Vision Analyzer 2 software (Brain Products, Gilching, Germany). Data were re‐referenced to the average of the two mastoids and high‐pass (0.1 Hz) and low‐pass (30 Hz) filtered. Standard eyeblink and ocular corrections were performed (Miller et al., 1988) and semiautomated artifact rejection procedures removed artifacts with the following criteria: voltage step of more than 50 μV between sample points, a voltage difference of 300 μV within a trial, and a maximum voltage difference of less than 0.5 μV within 100 ms intervals. Additional artifacts were removed using visual inspection. Data were baseline corrected using the 100 ms interval prior to feedback. ERPs were averaged across win and loss trials, and the RewP was scored as the mean amplitude 250–350 ms (based on previous research [Proudfit, 2015] and visualization of the scalp topography) following feedback at frontal site FCz, where the win minus loss difference was maximal (Figure 1). The mean number of artifact‐free trials for FCz for each condition were 19.14 (*SD* = 1.95) for win trials and 19.15 (*SD* = 1.92) for loss trials. The split‐half reliability Spearman–Brown coefficient for win trials was 0.78 and for loss trials was 0.82. The RewP is usually quantified as the win minus loss difference score (see Proudfit, 2015), with more positive values for the difference score indicating greater reactivity to reward. However, recent evidence suggests that residuals provide a more reliable ERP measure (see Meyer et al., 2017). Therefore, we used residual RewP scores, which were calculated by regressing Loss on Win trials for RewP‐Win residual scores and by regressing Win on Loss trials for RewP‐Loss residual scores. The split‐half reliability Spearman–Brown coefficient for Win‐residual was 0.69 and for Loss‐residual was 0.68.

### 2.5 fMRI data acquisition and preprocessing

An eyes‐open resting‐state functional MRI scan was collected for 8 minutes on a 3 T GE Discovery System (General Electric Healthcare; Waukesha, WI) with an 8‐channel head coil. Functional images were acquired using a gradient‐echo echo‐planar images (2 s TR, 25 ms TE, 82° flip, 64 × 64 matrix, 200 mm FOV, 3 mm slice thickness, 0 mm gap, with 44 axial slices). Imaging data were inspected and individuals with >2 mm displacement or rotation in any direction were not included in the analysis. The remaining subjects met criteria for high quality and scan stability. IP and HC did not significantly differ in peak movement (IP: *M* = 0.34 mm, *SD* = 0.31 mm; HC: *M* = 0.37 mm, *SD* = 0.26 mm), mean movement, or variability (*p*‐values > .05). Preprocessing of fMRI data was conducted using Statistical Parametric Mapping software (SPM8, Wellcome Department of Imaging Neuro‐Science, London, UK). Four initial volumes from each resting‐state run were discarded to allow for T1 equilibration effects. Images were spatially realigned, slice‐time corrected, warped to Montreal Neurological Institute (MNI) space using the participant's mean functional image, resampled to 2 mm^3^ voxels, and smoothed (8 mm^3^ FWHM Gaussian kernel). The effects of nuisance covariates (time‐series predictors for global signal, white matter, cerebrospinal fluid, and movement parameters including the first derivative), obtained during realignment to account for motion‐related effects in blood‐oxygen level‐dependent (BOLD) signal, were regressed from the data following the implemented anatomical component‐based noise correction method (aCompCor; Behzadi et al., 2007). The data were then bandpass filtered to 0.01–0.09 Hz.

### 2.6 Statistical analysis

Analyses were performed using SPM8 and SPSS 24.0 (IBM). Connectivity analyses were performed using the functional connectivity (CONN) toolbox (Whitfield‐Gabrieli & Nieto‐Castanon, 2012; www.nitrc.org/projects/conn) for statistical parametric mapping software (SPM8: Wellcome Trust Centre for Neuroimaging, London, UK).

#### 2.6.1 Functional connectivity relationship with the RewP‐Win and loss residuals

**FIGURE 2:**
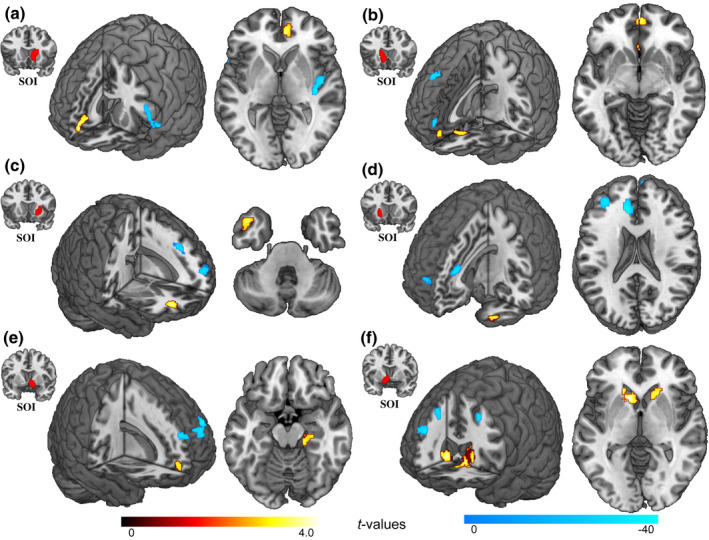
Relationships between RewP‐Win and resting‐state functional connectivity. Panel a shows that greater RewP‐Win residual was related to greater right caudate‐mPFC connectivity, decreased right caudate‐right posterior insula connectivity, and decreased right caudate‐left inferior frontal gyrus (IFG) connectivity (*p* < .05, corrected). Panel b shows that greater RewP‐Win residual was related to greater left caudate‐mPFC connectivity, greater left caudate‐mOFC connectivity, and decreased left caudate‐superior medial frontal gyrus (MFG) connectivity (*p* < .05, corrected). Panel c shows that greater RewP‐Win residual was related to greater right putamen‐mPFC connectivity, greater right putamen‐left superior temporal gyrus connectivity, and decreased right putamen‐left superior MFG connectivity (*p* < .05, corrected). Panel d shows that greater RewP‐Win residual was related to greater left putamen‐left superior temporal gyrus connectivity, decreased left putamen‐dorsal anterior cingulate cortex (dACC) connectivity, and decreased left putamen‐bilateral superior MFG connectivity (*p* < .05, corrected). Panel e shows that greater RewP‐Win residual was related to greater right NAcc‐right parahippocampal gyrus connectivity and decreased right NAcc‐bilateral superior MFG connectivity (*p* < .05, corrected). Panel f shows that greater RewP‐Win residual was related to greater left NAcc‐bilateral caudate connectivity, decreased left NAcc‐right superior MFG connectivity, and decreased left NAcc‐bilateral MFG (*p* < .05, corrected).

**FIGURE 3:**
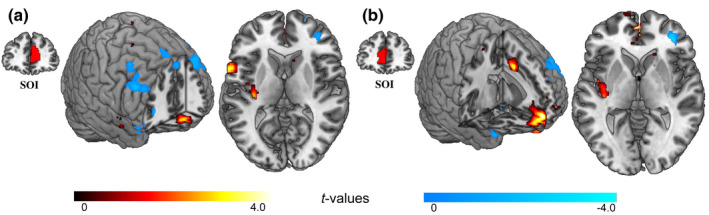
Relationships between RewP‐Loss and resting‐state functional connectivity. Panel a shows greater RewP‐Loss residual was related to greater right ACC‐mOFC, greater right ACC‐left precentral gyrus, decreased right ACC‐right lateral MFG, and decreased right ACC‐left superior medial frontal gyrus. Panel b shows greater RewP‐Loss residual was related to greater left ACC‐mOFC, greater left ACC‐MCC, greater left ACC‐left insula connectivity, decreased bilateral ACC‐right lateral MFG, and decreased left ACC‐right IFG connectivity.

Seed‐based connectivity analyses were performed by calculating the temporal correlation between BOLD signals from the striatum, including bilateral caudate, putamen, and NAcc seeds to all other voxels in the brain. The caudate and putamen seeds of interest (SOIs) were defined via the AAL atlas and created using MARINA (http://www.bion.de/Marina.html; Walter et al., 2003; see Figure 2). The NAcc SOI was anatomically predefined based on prior PET studies (Martinez et al., 2003, 2005; Figure 2). Visual inspection, using the Check Reg tool in SPM, showed the NAcc ROI was nearly identical to the AAL3 NAcc anatomical ROI. The ACC SOIs were defined by the CONN toolbox default atlas (see Figure 3). First level correlation maps were created for all subjects. At the second level, RewP‐Win residual values were regressed onto connectivity maps for right and left caudate, putamen, and NAcc seeds separately with group (IP, HC) as a covariate. RewP‐Loss residual values were regressed onto connectivity maps for right and left ACC seeds separately. Group differences in functional connectivity results from these models are reported in the supplement. Additional second‐level models were run for each seed with group and the interaction of group and ERP (RewP‐Win or Loss residuals) as covariates.

Given our strong a priori hypotheses regarding limbic and PFC connections, all results were restricted to a pre‐determined anatomical mask. This PFC and limbic mask consisted of the entire prefrontal cortex, and many limbic and subcortical areas including the temporal pole, anterior cingulate and paracingulate, posterior cingulate, hippocampus, parahippocampal gyrus, insula, amygdala, caudate, putamen, pallidum, and thalamus. As a null hypothesis, we also examined results from the left primary visual cortex. Due to concerns about high rates of false positives with lenient significance thresholds and following recent guidelines (Eklund et al., 2016; Woo et al., 2014), neural activity from task effects was considered significant if it exceeded correction of multiple comparisons across the PFC and limbic mask (volume = 575,248 mm^3^) using cluster‐based significant thresholding to adjust for multiple comparisons within the search volume using Monte Carlo simulations (10,000 iterations) performed with the most up‐to‐date version of 3dClustSim, an adaptation of AlphaSim (https://afni.nimh.nih.gov/pub/dist/doc/program_help/3dClustSim.html) in AFNI (19.1.06). The mixed autocorrelation function was utilized to give an accurate estimation of non‐Gaussian noise structure (Cox et al., 2017). Significance at corrected *α* < .05 and a voxel threshold of *p* < .005 yielded a minimum cluster size of at least 60 contiguous voxels (volume = 480 mm^3^). In order to show the distribution of activation data in a scatterplot, we extracted parameter estimates/*β*‐weights (arbitrary units [a.u.]) from a 10 mm radius sphere surrounding the peak activation. Functional connectivity related to the RewP‐ Win and Loss residuals were not significantly related to peak movement (*p*‐values > .05).

## 3 RESULTS

### 3.1 Relationship between functional connectivity and the RewP‐Win residual among all participants

Greater RewP‐Win residual was related to greater bilateral caudate‐medial prefrontal cortex (mPFC) connectivity (Table 2; right caudate seed Figure 2a; left caudate seed Figure 2b) and greater left caudate‐medial orbitofrontal cortex (mOFC) connectivity (Table 2; Figure 2b). Similarly, greater RewP‐Win residual was associated with greater bilateral putamen‐left superior temporal gyrus connectivity (Table 2; right putamen seed Figure 2c; left putamen seed Figure 2d). In addition, greater RewP‐Win residual was related to greater right NAcc‐right parahippocampal gyrus connectivity (Table 2; Figure 2e), greater left NAcc‐left caudate connectivity (Table 2; Figure 2f), and greater left NAcc‐right caudate connectivity (Table 2; Figure 2f). No results were significant for the left primary visual cortex, in line with our null hypothesis.

**TABLE 2:** Regions demonstrating functional connectivity relationship with the RewP‐Win residual

Region	Voxels	*Z* score	Peak coordinates (MNI)		
	(k)		*x*	*y*	*z*
*Positively associated with RewP‐Win*					
Right caudate seed					
mPFC	138	3.56	8	54	−4
Left caudate seed					
mPFC	63	3.49	2	52	−6
mOFC	65	3.25	−4	24	−14
Right putamen seed					
Left superior temporal gyrus	95	3.41	−44	14	−32
Left putamen seed					
Left superior temporal gyrus	76	3.42	−42	14	−30
Right NAcc seed					
Right parahippocampal gyrus	65	3.51	18	−26	−18
Left NAcc seed					
Left caudate	256	3.98	−10	18	−2
Right caudate	94	3.62	16	22	−2
*Negatively associated with RewP‐Win*					
Right caudate seed					
Right posterior insula	173	4.09	40	−18	4
Left IFG	84	3.62	−62	12	6
Left caudate seed					
Right superior MFG (BA 8)	174	3.76	18	36	48
Right superior MFG (BA 10)	127	3.75	8	60	10
Right putamen seed					
Left superior MFG	72	3.29	−10	64	22
Left putamen seed					
dACC	116	4.16	−12	34	20
Right lateral MFG	111	3.88	−36	44	24
Right superior MFG	75	3.50	8	64	16
Left superior MFG	120	3.15	−24	34	38
Right NAcc seed					
Left superior MFG	96	3.62	−30	54	34
Right superior MFG	91	3.05	16	62	34
Left NAcc seed					
Right superior MFG	69	3.35	14	56	34
Left MFG	75	3.20	−26	28	46
Right MFG	141	3.11	12	36	48

Abbreviations: IFG, inferior frontal gyrus; MFG, middle frontal gyrus; MNI, Montreal Neurological Institute; mOFC, medial orbitofrontal cortex; mPFC, medial prefrontal cortex; RewP, reward positivity.

On the other hand, greater RewP‐Win residual was related to decreased right caudate‐right posterior insula connectivity (Table 2; Figure 2a), decreased right caudate‐left inferior frontal gyrus (IFG) connectivity (Table 2; Figure 2a), and decreased left caudate‐superior medial frontal gyrus (MFG) connectivity (Table 2; Figure 2b). Greater RewP‐Win residual was associated with decreased right putamen‐left superior MFG connectivity (Table 2; Figure 2c), decreased left putamen‐dorsal anterior cingulate cortex (dACC) connectivity (Table 2; Figure 2d), and decreased left putamen‐bilateral superior MFG connectivity (Table 2; Figure 2d). Furthermore, greater RewP‐Win residual was related to decreased right NAcc‐bilateral superior MFG connectivity (Table 2; Figure 2e), decreased left NAcc‐right MFG connectivity (Table 2; Figure 2f).

### 3.2 Relationship between functional connectivity and the RewP‐Loss residual among all participants

Greater RewP‐Loss residual was related to greater bilateral ACC‐mOFC, right ACC‐left precentral gyrus, left ACC‐MCC, and left ACC‐left insula connectivity (Table 3; Figure 3). Conversely, greater RewP‐Loss residual was related to decreased bilateral ACC‐right lateral MFG, right ACC‐left superior medial frontal gyrus, and left ACC‐right IFG connectivity (see Table 3; Figure 3). No results were significant for the left primary visual cortex, in line with our null hypothesis.

**TABLE 3:** Regions demonstrating functional connectivity relationship with the RewP‐Loss residual

Region	Voxels	*Z* score	Peak coordinates (MNI)		
	(k)		*x*	*y*	*z*
*Positively associated with RewP‐Loss*					
Right ACC					
mOFC	262	4.03	0	52	−10
Left precentral gyrus	95	3.70	−62	8	8
Left ACC					
mOFC	611	4.25	−6	−52	−12
MCC	84	3.53	−6	10	36
Left insula	130	3.44	−34	−20	8
*Negatively associated with RewP‐Loss*					
Right ACC					
Left superior medial frontal gyrus	75	3.52	−4	58	42
Right lateral MFG	76	3.34	42	20	32
Left ACC					
Right lateral MFG to IFG	300	3.73	44	22	32
Right IFG	77	3.60	36	42	4

Abbreviations: ACC, anterior cingulate cortex; IFG, inferior frontal gyrus; MCC, midcingulate cortex; MFG, middle frontal gyrus; MNI, Montreal Neurological Institute; mOFC, medial orbitofrontal cortex; mPFC, medial prefrontal cortex; RewP, reward positivity.

### 3.3 Interaction between the group and the RewP‐Win residual on functional connectivity

**FIGURE 4:**
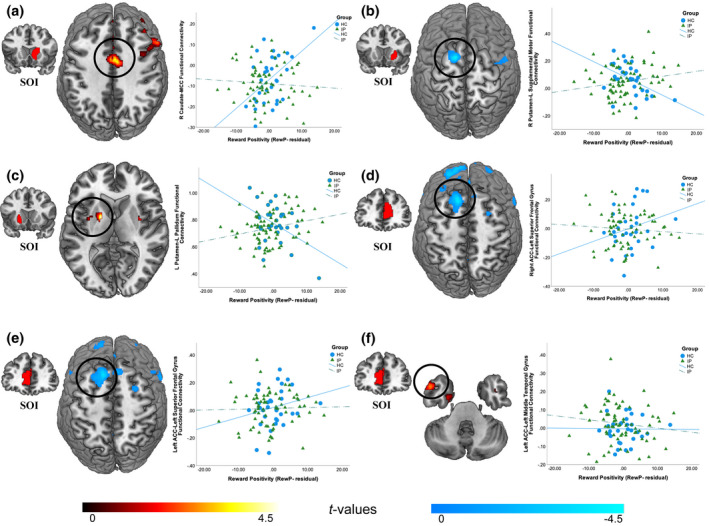
Interaction between group and ERPs on resting‐state functional connectivity. Panel a shows that greater RewP‐Win residual was related to greater right caudate‐MCC connectivity (MNI peak activation [4, −2, 40]; activation circled) for HC, but not for IP in order to show the distribution of the data. Panel b shows that greater RewP‐Win residual was related to decreased right putamen‐left supplemental motor area connectivity (MNI peak activation [−14, 2, 66]; activation circled) for HC, but not for IP in order to show the distribution of the data. Panel c shows that greater RewP‐Win residual was related to decreased left putamen‐left pallidum connectivity (MNI peak activation [−18, 0, 8]; activation circled) for HC, while greater RewP‐Win residual was related to greater left putamen‐left pallidum connectivity for IP in order to show the distribution of the data. Panel d shows that greater RewP‐Loss residual was related to decreased right ACC‐left superior frontal gyrus connectivity (MNI peak activation [−12, 22, 52]; activation circled) for HC, but not for IP in order to show the distribution of the data. Panel e shows that greater RewP‐Loss residual was related to decreased left ACC‐left superior frontal gyrus connectivity (MNI peak activation [−12, 22, 52]; activation circled) for HC, but not for IP in order to show the distribution of the data. Panel f shows that greater RewP‐Loss residual was related to increased left ACC‐middle temporal gyrus connectivity (MNI peak activation [−48, 14, −38]; activation circled) for IP, but not for HC in order to show the distribution of the data.

Regions demonstrating an interaction between group and the RewP‐Win residual on functional connectivity are shown in Table 4. We ran follow‐up analyses of the simple slopes for significant interactions from the SPM models to determine the direction of the results. Results of the simple slopes analyses showed that greater RewP‐Win residual was related to greater right caudate‐MCC connectivity for HC (*β* = .25, *p* = .04), but not for IP, *β* = −.07, *p* = .55 (Figure 4a). Similarly, greater RewP‐Win residual was related to greater right caudate‐right lateral MFG/IFG connectivity for HC (*β* = .41, *p* = .02), but not for IP, *β* = −.14, *p* = .26. Furthermore, greater RewP‐Win residual was related to decreased right putamen‐left supplemental motor area connectivity for HC (*β* = −.48, *p* = .006), but not for IP, *β* = .19, *p* = .11 (Figure 4b). In addition, greater RewP‐Win residual was related to decreased left putamen‐left pallidum connectivity for HC (*β* = −.44, *p* = .01), while greater RewP‐Win residual was related to greater left putamen‐left pallidum connectivity for IP, *β* = .25, *p* = .04 (Figure 4c).

**TABLE 4:** Interaction effects of group and RewP‐Win and Loss residuals on functional connectivity

Region	Voxels	*Z* score	Peak coordinates (MNI)		
	(k)		*x*	*y*	*z*
*Positive interaction*					
Right caudate seed—RewP‐Win					
*n/a*	–	–	–	–	–
Left caudate seed—RewP‐Win					
*n/a*	–	–	–	–	–
Right putamen seed—RewP‐Win					
Left supplemental motor	134	4.05	−14	2	66
Left putamen seed—RewP‐Win					
Right hippocampus	94	3.79	32	−20	−8
Left pallidum	69	3.72	−18	0	8
Right NAcc seed—RewP‐Win					
*n/a*	–	–	–	–	–
Left NAcc seed—RewP‐Win					
*n/a*	–	–	–	–	–
Right ACC seed—RewP‐Loss					
Left parahippocampal gyrus	148	4.13	−30	−32	−14
Left superior MFG	451	4.03	−12	44	34
Left superior frontal gyrus	209	3.92	−12	22	52
Left temporal pole	146	3.76	−52	12	−36
Left IFG	149	3.38	−56	22	2
Left ACC seed—RewP‐Loss					
Left parahippocampal gyrus	219	4.18	−30	−32	−14
Left superior frontal gyrus	210	3.78	−12	22	52
Left middle temporal gyrus	85	3.64	−48	14	−38
Left IFG	77	3.16	−54	24	2
*Negative interaction*					
Right caudate seed—RewP‐Win					
MCC	228	3.78	4	−2	40
Right lateral MFG to IFG	78	3.18	58	22	26
Left caudate seed—RewP‐Win					
*n/a*	–	–	–	–	–
Right putamen seed—RewP‐Win					
*n/a*	–	–	–	–	–
Left putamen seed—RewP‐Win					
*n/a*	–	–	–	–	–
Right NAcc seed—RewP‐Win					
MCC	66	3.31	2	−2	42
Left NAcc seed—RewP‐Win					
Left superior temporal gyrus	66	3.27	−22	14	−32
Right ACC—RewP‐Loss					
Left paracentral gyrus	148	3.68	−14	−24	70
Left ACC—RewP‐Loss					
Left paracentral gyrus	101	3.47	−16	−20	78

Abbreviations: ACC, anterior cingulate cortex; IFG, inferior frontal gyrus; MFG, middle frontal gyrus; MNI, Montreal Neurological Institute; mPFC, medial prefrontal cortex; RewP, reward positivity.

Although the interaction between RewP‐Win residual and group was significant for left putamen‐right hippocampus connectivity, right NAcc‐MCC connectivity, and left NAcc‐ left superior temporal gyrus connectivity in SPM (see Table 4), analyses of the simple slopes found no significant simple slopes.

### 3.4 Interaction between the group and the RewP‐Loss residual on functional connectivity

Regions demonstrating an interaction between group and the RewP‐Loss residual on functional connectivity are shown in Table 4. Results of the simple slopes analyses showed that greater RewP‐Loss residual was related to decreased right ACC‐left superior frontal gyrus connectivity for HC (*β* = −.44, *p* = .01), but not for IP, *β* = .23, *p* = .052 (Figure 4d). In addition, greater RewP‐Loss residual was related to decreased left ACC‐left superior frontal gyrus connectivity for HC (*β* = −.41, *p* = .02), but not for IP, *β* = .11, *p* = .37 (Figure 4e). On the other hand, greater RewP‐Loss residual was related to increased left ACC‐middle temporal gyrus connectivity for IP (*β* = .26, *p* = .03), but not for HC *β* = −.20, *p* = .29 (Figure 4f).

Although the interaction between RewP‐Loss residual and group was significant for right ACC‐left parahippocampal gyrus, right ACC‐left superior MFG, right ACC‐left temporal pole, right ACC‐left IFG, right ACC‐left paracentral gyrus, left ACC‐left parahippocampal gyrus, left ACC‐left IFG, and left ACC‐left paracentral gyrus in SPM (see Table 4), follow‐up analyses of the simple slopes found no significant simple slopes.

## 4 DISCUSSION

The current study sought to examine whether the RewP (Win and Loss residuals), a neurophysiological index of reward responsiveness, was associated with resting‐state fMRI connectivity in reward‐related brain regions among adults with and without internalizing psychopathology. Results revealed that individuals with greater RewP‐Win residual amplitude demonstrated greater functional connectivity of several reward‐related brain regions. We also found that individuals with greater RewP‐Win residual amplitude showed less connectivity between striatal reward regions and regions involved in cognitive control and sensorimotor processing, including the superior MFG, IFG, dACC, and posterior insula. In addition, greater RewP‐Loss residual was related to greater connectivity between the ACC and regions involved in reward value (mOFC), loss processing (insula), motor control (precentral gyrus), and error processing (MCC), but decreased connectivity between the ACC and regions involved in cognitive control, including the lateral MFG, superior MFG, and IFG. Together, these findings indicate that connectivity of the mesocorticolimbic system may be an important individual difference factor across diagnostic groups in determining how individuals respond to the attainment of reward and loss.

Our novel findings of a relationship between greater RewP‐Win residual and increased functional connectivity of brain reward regions indicates that individual differences in connectivity are associated with neurophysiological reward processing among IP and HC. Prior studies have found that the RewP (Win > Loss) may originate from the striatum (Foti et al., 2011) and that the RewP is related to BOLD and EEG activation of brain regions implicated in reward, including the striatum and mPFC during reward attainment among healthy individuals (Becker et al., 2014; Carlson et al., 2011; Foti et al., 2011; Gehring & Willoughby, 2002). In the current study, we have extended these previous studies by showing for the first time that increased resting‐state connectivity of these brain reward regions, especially between striatal regions and the mPFC, were related to a greater RewP‐Win residual. We also found that increased resting‐state connectivity between striatal regions and regions in the temporal lobe, including the superior temporal gyrus and the parahippocampal gyrus, were related to greater RewP‐Win residual. These temporal regions have been shown in previous neuroimaging studies to be related to reward (see Patel et al., 2013; Wang et al., 2014), especially in value representation, and are connected to the striatum via corticostriatal pathways (Haber, 2016). This suggests that individuals with greater coupling between brain reward regions involved in assessing the value of a reward (superior temporal gyrus) and regions involved in goal directed behavior (striatum) may find rewarding stimuli to be more salient and/or may have a greater motivation for rewards, and therefore these individuals show greater initial responsiveness to reward. Indeed, prior studies find that individuals with a larger RewP demonstrated increased approach motivation and greater performance monitoring, especially when individuals believe that their effort led to the reward(s) (Harmon‐Jones et al., 2020; Threadgill & Gable, 2018). Taken together, our findings suggest that individuals who have greater coupling between brain reward regions involved in goal‐directed behavior and reward valuation may be more motivated for rewards and better able to monitor their performance at each stage of goal pursuit, which may generally result in better performance and increased their likelihood of reward attainment.

In addition, findings demonstrated that individuals with greater RewP‐Win residual showed decreased connectivity between striatal regions and the superior MFG, left IFG, dACC, and posterior insula. The superior MFG, left IFG, and dACC are regions implicated in cognitive control (Cole & Schneider, 2007; Li et al., 2013). Thus, individuals with decreased connectivity between striatal reward regions and these prefrontal cognitive control regions may have less intrinsic connectivity supporting top‐down control of rewarding stimuli and therefore show greater RewP‐Win residual during reward processing. The posterior insula has strong connections with sensorimotor areas, including the striatum, and is thought to be involved in monitoring pain and interoceptive processing (Strigo & Craig, 2016). Our findings of an association between greater RewP‐Win residual and decreased connectivity between the right caudate and the right posterior insula connectivity suggest that individuals who are more responsive to rewards may have less intrinsic connectivity supporting sensorimotor processing. As such, it is possible that when presented with a reward, these individuals focus more on the external rewarding stimuli and less on internal bodily states.

To better understand how individual differences in loss responsivity are related to rs‐fMRI connectivity in loss‐related brain regions, we also examined the association between the RewP‐Loss residual and rs‐fMRI. We used ACC seeds for relationships between the RewP‐Loss residual and rs‐fMRI, based on previous source localization studies have identified that the FN (Loss > Win) may originate from the ACC (Dehaene et al., 1994; Miltner et al., 1997), a region involved in error processing and cognitive control. We found that greater RewP‐Loss residual was related to greater connectivity between the ACC and regions involved in reward value (mOFC) (Rolls et al., 2020), loss processing (insula) (Dugre et al., 2018), reward or outcome information (MCC) (Rolls, 2019), and motor control (precentral gyrus) (Catani, 2019). On the other hand, greater RewP‐Loss residual was related to decreased connectivity between the ACC and regions involved in cognitive control, including the lateral MFG, superior MFG, and IFG. It is interesting that we observed decreased rs‐fMRI connectivity was related to both striatal seeds for greater RewP‐Win residual and ACC seeds for greater RewP‐Loss residual. As such, individuals with decreased connectivity between striatal, as well as ACC regions and prefrontal cognitive control regions may have less intrinsic connectivity supporting top‐down control of not only rewarding stimuli, but also loss stimuli, which may result in enhanced RewP‐Win residual during reward responsivity and enhanced RewP‐Loss residual during loss responsivity.

The current study also sought to extend prior research by exploring whether associations between the RewP‐Win and Loss residuals and rs‐fMRI were comparable across IP and HC. Interestingly, the relationship between RewP and resting‐state connectivity were generally consistent across IP and HC. This indicates that intrinsic connectivity between reward‐related brain regions and fronto‐limbic regions is an important individual difference factor in reward processing that generally does not differ between psychiatrically‐healthy individuals and individuals with internalizing psychopathology. However, we found a few exceptions, in which IP status moderated the relationship between reward processing and resting‐state connectivity. Specifically, we found greater RewP‐Win residual was associated with decreased right putamen‐left supplemental motor area connectivity for HC, but not for IP. In addition, greater RewP‐Win residual was related to decreased left putamen‐left pallidum connectivity for HC, while IP demonstrated the opposite pattern‐ greater RewP‐Win residual was related to greater left putamen‐left pallidum connectivity. Furthermore, greater RewP‐Win residual was related to greater right caudate‐MCC connectivity and greater right caudate‐right lateral MFG/IFG connectivity for HC, but not for IP. Therefore, it is possible that for healthy adults, individuals with greater reward responsivity demonstrate slight increases in resting‐state connectivity between reward regions and regions involved in decision‐making and response inhibition, as well as slight decreases between reward regions and between reward and motor regions. IP, however, showed a different pattern. For IP, individuals with greater reward responsivity only demonstrate slight increases in resting‐state connectivity between reward regions. These patterns may relate to dispositional characteristics such as trait impulsivity, a risk factor of self‐harm in internalizing conditions (Rawlings et al., 2015), which has been shown to predict RewP (Ait Oumeziane & Foti, 2016). It also suggests reward‐related connectivity involving cognitive control may be useful in classifying diagnostic status (e.g., IP vs. HC), which has inferences for treatment. When examining how IP status moderated the relationship between the RewP‐Loss residual and rs‐fMRI connectivity, we found several significant interactions (see Table 4); however, follow‐up analyses of the simple slopes found that only two significant group differences in the relationship between the RewP‐Loss residual and rs‐fMRI connectivity. First, greater RewP‐Loss residual was related to decreased bilateral ACC‐left superior frontal gyrus connectivity for HC, but not for IP. Therefore, HC show the same pattern as the overall findings described above, such that healthy individuals with decreased connectivity between ACC loss‐related regions and prefrontal cognitive control regions may have less intrinsic connectivity supporting top‐down control of loss stimuli, which may result in enhanced RewP‐Loss residual during loss responsivity. On the other hand, this is not always the case for IP, who do not show a relationship between ACC‐left superior frontal gyrus connectivity and RewP‐Loss residual. It is possible that intrinsic connectivity supporting top‐control of loss stimuli is not as directly related to loss responsivity for IP. Second, greater RewP‐Loss residual was related to increased left ACC‐ middle temporal gyrus connectivity for IP, but not for HC.

Therefore, it is possible that for healthy adults, individuals with greater reward responsivity demonstrate slight increases in resting‐state connectivity between reward regions and regions involved in decision‐making and response inhibition, as well as slight decreases between reward regions and between reward and motor regions. For IP, individuals with greater reward responsivity only demonstrate slight increases in resting‐state connectivity between reward regions. These patterns may relate to dispositional characteristics such as trait impulsivity, a risk factor of self‐harm in internalizing conditions (Rawlings et al., 2015), which has been shown to predict RewP (Ait Oumeziane & Foti, 2016). It also suggests reward‐related connectivity involving cognitive control may be useful in classifying diagnostic status (e.g., IP vs. HC), which has inferences for treatment.

It is important to note that we found group differences in rs‐fMRI connectivity patterns (see Supplement). Overall, compared to HC, IP demonstrated a pattern of decreased frontostriatal rs‐fMRI connectivity, as well as decreased ACC‐MFG and ACC‐MCC rs‐fMRI connectivity. This is in line with some previous studies that found rs‐fMRI frontostriatal hypoconnectivity among individuals with depression (see Li et al., 2018). There have been fewer studies examining rs‐fMRI connectivity among individuals with anxiety disorders; however, one study found decreased resting‐state functional connectivity between reward regions including the nucleus accumbens and the ventromedial prefrontal cortex (vmPFC) among individuals with social anxiety disorder versus healthy controls (Manning et al., 2015). On the other hand, we did find some regions that showed increased rs‐fMRI connectivity among IP compared to HC, including increased rs‐fMRI connectivity between the dorsal striatum (i.e., caudate, putamen) and regions involved in cognitive control (superior MFG) and motor/sensory processing (paracentral lobule), as well as between the ACC and regions involved in motor/sensory processing and integration (paracentral lobule, left superior temporal gyrus). More studies of individuals with depression, anxiety, and comorbid depression and anxiety are needed to better understand contributions to region‐specific decreases and increases in rs‐fMRI connectivity among individuals with internalizing disorders.

Although the current study has several strengths, including a large sample of unmedicated adults with and without internalizing psychopathology, which allowed for comorbid internalizing psychopathology, the study also has important limitations. Data collected with EEG and fMRI were not simultaneous, therefore, possible confounds related to temporal factors cannot be ruled out. Participants had a range of internalizing psychopathology, but some principal diagnoses were under‐represented (i.e., panic disorder, PTSD) and more IP than HC made up the sample. In addition, given the high prevalence of comorbidity in the sample, we did not have enough power to examine if the relationship between RewP‐Win and Loss residuals and resting‐state connectivity differed by specific diagnoses. Thus, future studies should explore whether the degree of association between the RewP‐Win and Loss residuals and rs‐fMRI differs in a sample of healthy controls and IP without comorbidity. Furthermore, we did not find a group difference in wins, losses, or the RewP between IPs and HC. Although there is substantial evidence that depression risk is characterized by a blunted RewP (see Kujawa & Burkhouse, 2017; Proudfit, 2015), there is mixed evidence as to whether the RewP is associated with anxiety. Few studies have examined whether the RewP is disrupted among individuals with comorbid depression and anxiety, even though comorbidity of these disorders is extremely common. Therefore, it is possible that we did not see group differences in the RewP (or FN) due to the high prevalence of comorbid depression and anxiety in the current study. This will be important for future studies to examine.

Despite limitations, results provide preliminary evidence that the RewP, a neurophysiological marker of initial response to reward, is associated with functional connectivity of brain regions implicated in reward during resting‐state fMRI. These findings indicate that individuals who are more responsive to rewards have greater connectivity between reward‐related brain regions. We also found that individuals with greater RewP‐Win residual showed decreased connectivity between striatal regions and regions involved in cognitive control and sensorimotor processing. Furthermore, we found that greater RewP‐Loss residual was related to greater connectivity between the ACC and regions involved in reward and loss processing and motor control, but decreased connectivity between the ACC and regions involved in cognitive control. Moreover, our findings were generally consistent across IP and HC though certain results were unique to IP. Therefore, connectivity of the mesocorticolimbic system appears to be an important individual difference factor related to how individuals respond to the attainment of reward and loss across individuals with and without internalizing psychopathology.

## AUTHOR CONTRIBUTIONS

**Natania A Crane:** Conceptualization; data curation; formal analysis; investigation; visualization; writing – original draft. **Katie Burkhouse:** Formal analysis; methodology; visualization; writing – review and editing. **Stephanie M. Gorka:** Formal analysis; writing – review and editing. **Heide Klumpp:** Project administration; writing – review and editing. **Luan Phan:** Funding acquisition; investigation; project administration; resources; writing – review and editing.

## FUNDING INFORMATION

This publication was funded by the National Institute of Mental Health (NIMH) (R01MH101497, PI: KLP) and supported through the National Institutes of Health (NIH) through the UIC Center for Clinical and Translational Science (CCTS) (UL1TR002003) and the UIC Center for Magnetic Resonance Research (CMRR) (S10RR028898). NAC, KLB, and HK were supported by NIMH (T32MH067631, PI: Rasenick; K23MH113793, PI: KLB; and R01MH112705, PI: HK, respectively) and the National Institute on Drug Abuse (NIDA; K23DA048132, PI: NAC). SMG was supported by the National Institute on Alcohol Abuse and Alcoholism (NIAAA) (K23AA025111, PI: SMG). Its contents are solely the responsibility of the authors and do not necessarily represent the official views of NIMH, NIDA, NIAAA, or the National Institutes of Health

## CONFLICT OF INTEREST

The authors declare no conflicts of interest.

## Supporting information


**TABLE S1**. Group differences in functional connectivity
**FIGURE S1**. Group differences in resting‐state functional connectivity with caudate seeds of interest. Panel a shows that IP had greater right caudate‐paracentral lobule and right caudate‐left superior MFG connectivity compared to HC (in red/yellow), while IP demonstrated less right caudate‐right lateral MFG, right caudate‐superior medial MFG, right caudate‐MCC, right caudate‐right anterior insula, and right caudate‐left lateral OFC connectivity compared to HC (in blue/teal). Panel b shows that IP showed greater left caudate‐paracentral lobule and left caudate‐left superior MFG connectivity compared to HC (in red/yellow), while IP demonstrated less left caudate‐right lateral MFG, left caudate‐superior medial MFG, and left caudate‐MCC compared to HC (in blue/teal)
**FIGURE S2**. Group differences in resting‐state functional connectivity with putamen seeds of interest. Panel a shows that IP demonstrated greater right putamen‐superior MFG connectivity to HC (in red/yellow), while IP demonstrated less right caudate‐right lateral MFG, right caudate‐superior medial MFG, right caudate‐MCC, right caudate‐right anterior insula, and right caudate‐left lateral OFC connectivity compared to HC (in blue/teal). Panel b shows that IP demonstrated greater left putamen‐superior MFG and left putamen‐left paracentral lobule connectivity to HC (in red/yellow), while IP demonstrated less left putamen‐right OFC connectivity compared to HC (in blue/teal)
**FIGURE S3**. Group differences in resting‐state functional connectivity with NAcc seeds of interest. IP demonstrated less right NAcc‐medial MFG/supplemental motor, right NAcc‐right OFC, and right NAcc‐right lateral superior MFG connectivity compared to HC (in blue/teal). There were no significant group differences in left NAcc connectivity
**FIGURE S4**. Group differences in resting‐state functional connectivity with ACC seeds of interest. Panel a shows that IP demonstrated greater right ACC‐left superior temporal gyrus and right ACC‐bilateral paracentral gyrus connectivity compared to HC (in red/yellow), while IP demonstrated less right ACC‐MFG and right ACC‐lateral MFG connectivity compared to HC (in blue/teal). Panel b shows that IP demonstrated less left ACC‐MFG and left ACC‐MCC connectivity compared to HC (in blue/teal)
