# Supplementary material for: Electrocortical measures of win and loss processing are associated with mesocorticolimbic functional connectivity: A combined ERP and rs‐fMRI study
Source: Psychophysiology. 2022 Jun 7;59(12):e14118. doi: 10.1111/psyp.14118 (PMC9643675; doi:10.1111/psyp.14118)
Supplement: Supplementary file 1 — TABLE S1. Group differences in functional connectivity FIGURE S1. Group differences in resting‐state functional connectivity with caudate seeds of interest. Panel a shows that IP had greater right caudate‐paracentral lobule and right caudate‐left superior MFG connectivity compared to HC (in red/yellow), while IP demonstrated less right caudate‐right lateral MFG, right caudate‐superior medial MFG, right caudate‐MCC, right caudate‐right anterior insula, and right caudate‐left lateral OFC connectivity compared to HC (in blue/teal). Panel b shows that IP showed greater left caudate‐paracentral lobule and left caudate‐left superior MFG connectivity compared to HC (in red/yellow), while IP demonstrated less left caudate‐right lateral MFG, left caudate‐superior medial MFG, and left caudate‐MCC compared to HC (in blue/teal) FIGURE S2. Group differences in resting‐state functional connectivity with putamen seeds of interest. Panel a shows that IP demonstrated greater right putamen‐superior MFG connectivity to HC (in red/yellow), while IP demonstrated less right caudate‐right lateral MFG, right caudate‐superior medial MFG, right caudate‐MCC, right caudate‐right anterior insula, and right caudate‐left lateral OFC connectivity compared to HC (in blue/teal). Panel b shows that IP demonstrated greater left putamen‐superior MFG and left putamen‐left paracentral lobule connectivity to HC (in red/yellow), while IP demonstrated less left putamen‐right OFC connectivity compared to HC (in blue/teal) FIGURE S3. Group differences in resting‐state functional connectivity with NAcc seeds of interest. IP demonstrated less right NAcc‐medial MFG/supplemental motor, right NAcc‐right OFC, and right NAcc‐right lateral superior MFG connectivity compared to HC (in blue/teal). There were no significant group differences in left NAcc connectivity FIGURE S4. Group differences in resting‐state functional connectivity with ACC seeds of interest. Panel a shows that IP demonstrated greater right ACC‐left [file PSYP-59-e14118-s001.docx]

**S1. Group differences in functional connectivity**

IP showed greater bilateral caudate-paracentral lobule and bilateral caudate-left superior MFG connectivity compared to HC (see Supplemental Table 1, Supplemental Figure 1). IP demonstrated greater bilateral putamen-superior MFG and left putamen-left paracentral lobule connectivity to HC (see Supplemental Table 1, Supplemental Figure 2). In addition, IP showed greater right ACC-left superior temporal gyrus and right ACC-bilateral paracentral gyrus connectivity compared to HC (see Supplemental Table 1, Supplemental Figure 4).

On the other hand, IP demonstrated less bilateral caudate-right lateral MFG, bilateral caudate-superior medial MFG, bilateral caudate-MCC, right caudate-right anterior insula, and right caudate-left lateral OFC connectivity compared to HC (see Supplemental Table 1, Supplemental Figure 1). IP showed less bilateral putamen-right OFC, right putamen-right lateral MFG/IFG, right putamen-IFG, and left putamen-left caudate connectivity compared to HC (see Supplemental Table 1, Supplemental Figure 2). In addition, IP showed less right NAcc-medial MFG/supplemental motor, right NAcc-right OFC, and right NAcc-right lateral superior MFG connectivity compared to HC (see Supplemental Table 1, Supplemental Figure 3). Further, IP demonstrated less bilateral ACC-MFG, right ACC-lateral MFG, and left ACC-MCC connectivity compared to HC (see Supplemental Table 1, Supplemental Figure 4).

| **Supplemental Table 1. Group Differences in Functional Connectivity** | | | | | | | |  | |
| --- | --- | --- | --- | --- | --- | --- | --- | --- | --- |
| **Region** | | | **Voxels** | | **Z score** | **Peak Coordinates (MNI)** | | | |
|  |  | | **(k)** | |  | **x** | **y** | | **z** |
| ***IP > HC*** | | | | |  |  |  | |  |
| **Right caudate seed** | | | |  |  |  |  |  | |
|  | Bilateral paracentral lobule | | | 301 | 3.40 | 8 | -32 | 64 | |
|  | Left superior MFG | | | 151 | 3.04 | -22 | 60 | 26 | |
| **Left caudate seed** | | | |  |  |  |  |  | |
|  | Bilateral paracentral lobule | | | 387 | 3.84 | 12 | -32 | 66 | |
|  | Left superior MFG | | | 185 | 3.28 | -8 | 60 | 30 | |
| **Right putamen seed** | | | |  |  |  |  |  | |
|  | Left superior MFG | | | 175 | 3.34 | -10 | 44 | 34 | |
| **Left putamen seed** | | | |  |  |  |  |  | |
|  | Left paracentral lobule | | | 150 | 3.40 | -6 | -16 | 72 | |
|  | Left superior frontal gyrus | | | 203 | 3.39 | -16 | 52 | 26 | |
| **Right NAcc seed** | | | |  |  |  |  |  | |
|  | | *n/a* | | -- | -- | -- | -- | -- | |
| **Left NAcc seed** | | | |  |  |  |  |  | |
|  | | *n/a* | | -- | -- | -- | -- | -- | |
| **Right ACC seed** | | | |  |  |  |  |  | |
|  | | Left superior temporal gyrus | | 62 | 3.44 | -32 | 6 | -28 | |
|  | | Bilateral paracentral gyrus | | 262 | 3.37 | 8 | -34 | 58 | |
| **Left ACC seed** | | | |  |  |  |  |  | |
|  | | *n/a* | | -- | -- | -- | -- | -- | |
| ***IP < HC*** | | | |  |  |  |  |  | |
| **Right caudate seed** | | | |  |  |  |  | |  |
|  | Right lateral MFG to IFG | | | 533 | 3.94 | 40 | 38 | | 24 |
|  | Superior medial MFG | | | 189 | 3.49 | 4 | 28 | | 44 |
|  | Right anterior insula | | | 147 | 3.34 | 52 | 18 | | -12 |
|  | Left lateral OFC | | | 60 | 3.33 | -26 | 36 | | -18 |
|  | Right MCC | | | 78 | 3.24 | 10 | -30 | | 38 |
| **Left caudate seed** | | | |  |  |  |  | |  |
|  | MCC | | | 166 | 3.57 | 8 | -32 | | 44 |
|  | Right lateral MFG | | | 117 | 3.56 | 42 | 12 | | 50 |
|  | Superior medial MFG | | | 88 | 3.55 | 6 | 34 | | 48 |
| **Right putamen seed** | | | |  |  |  |  | |  |
|  | Right lateral MFG to IFG | | | 121 | 3.37 | 40 | 38 | | 22 |
|  | Right OFC | | | 114 | 3.28 | 18 | 38 | | -26 |
|  | Right IFG | | | 88 | 3.04 | 48 | 6 | | 16 |
| **Left putamen seed** | | | |  |  |  |  | |  |
|  | Left caudate | | | 118 | 4.12 | -12 | 4 | | 22 |
|  | Right lateral OFC | | | 156 | 3.47 | 22 | 38 | | -20 |
| **Right NAcc seed** | | | |  |  |  |  | |  |
|  | Medial MFG/supplemental motor | | | 216 | 3.85 | 4 | 26 | | 44 |
|  | Right OFC | | | 70 | 3.37 | 22 | 40 | | -22 |
|  | Right lateral superior MFG | | | 106 | 3.17 | 38 | 16 | | 54 |
| **Left NAcc seed** | | | |  |  |  |  | |  |
|  | *n/a* | | | -- | -- | -- | -- | | -- |
| **Right ACC** | | | |  |  |  |  | |  |
|  | Right lateral MFG (BA 9) | | | 83 | 3.56 | 54 | 30 | | 34 |
|  | Right MFG | | | 72 | 3.10 | 34 | 24 | | 36 |
| **Left ACC** | | | |  |  |  |  | |  |
|  | MCC | | | 141 | 3.47 | -6 | -20 | | 32 |
|  | Left MFG (BA 9) | | | 98 | 3.43 | -32 | 18 | | 34 |
| *Note.* ACC= anterior cingulate cortex, HC= healthy controls, IFG= inferior frontal gyrus, IP= individuals with internalizing psychopathology, MCC= mid-cingulate cortex, MFG= middle frontal gyrus, MNI= Montreal Neurological Institute, mPFC= medial prefrontal cortex, and OFC= orbitofrontal cortex. | | | | | | | | | |

**
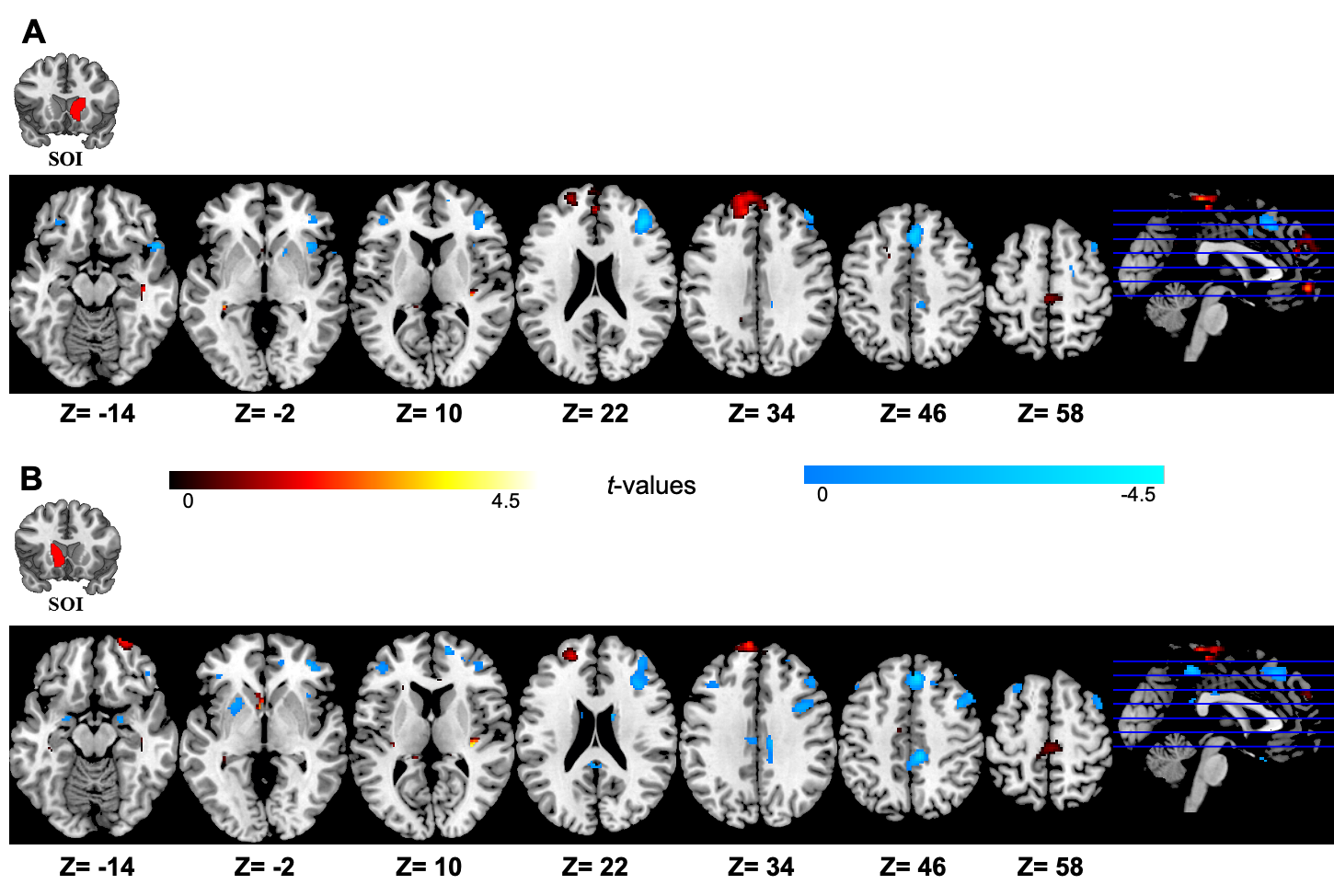
**

**Supplemental Figure 1.** Group Differences in Resting-state Functional Connectivity with Caudate Seeds of Interest. Panel A shows that IP had greater right caudate-paracentral lobule and right caudate-left superior MFG connectivity compared to HC (in red/yellow), while IP demonstrated less right caudate-right lateral MFG, right caudate-superior medial MFG, right caudate-MCC, right caudate-right anterior insula, and right caudate-left lateral OFC connectivity compared to HC (in blue/teal). Panel B shows that IP showed greater left caudate-paracentral lobule and left caudate-left superior MFG connectivity compared to HC (in red/yellow), while IP demonstrated less left caudate-right lateral MFG, left caudate-superior medial MFG, and left caudate-MCC compared to HC (in blue/teal).


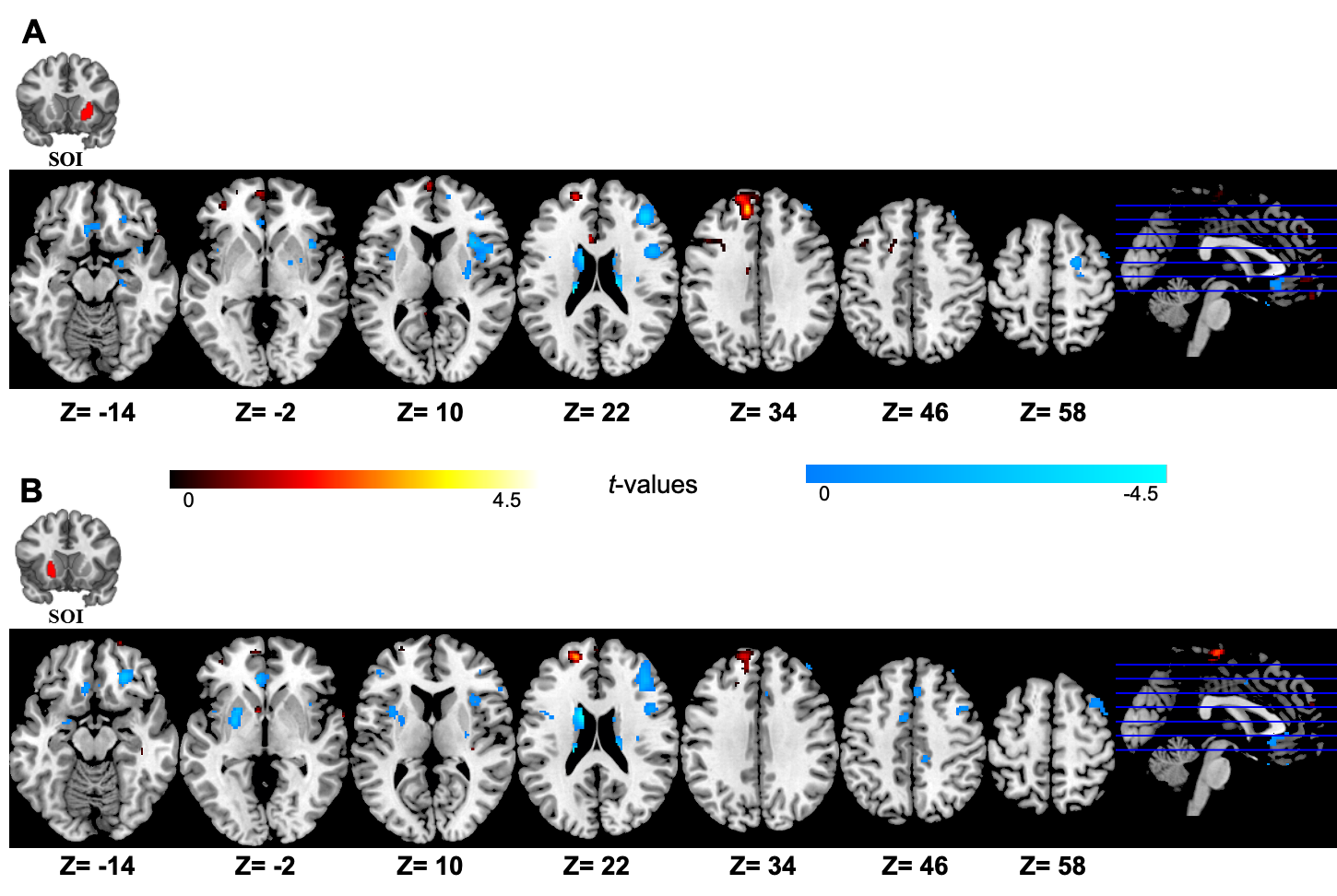


**Supplemental Figure 2.** Group Differences in Resting-state Functional Connectivity with Putamen Seeds of Interest. Panel A shows that IP demonstrated greater right putamen-superior MFG connectivity to HC (in red/yellow), while IP demonstrated less right caudate-right lateral MFG, right caudate-superior medial MFG, right caudate-MCC, right caudate-right anterior insula, and right caudate-left lateral OFC connectivity compared to HC (in blue/teal). Panel B shows that IP demonstrated greater left putamen-superior MFG and left putamen-left paracentral lobule connectivity to HC (in red/yellow), while IP demonstrated less left putamen-right OFC connectivity compared to HC (in blue/teal).


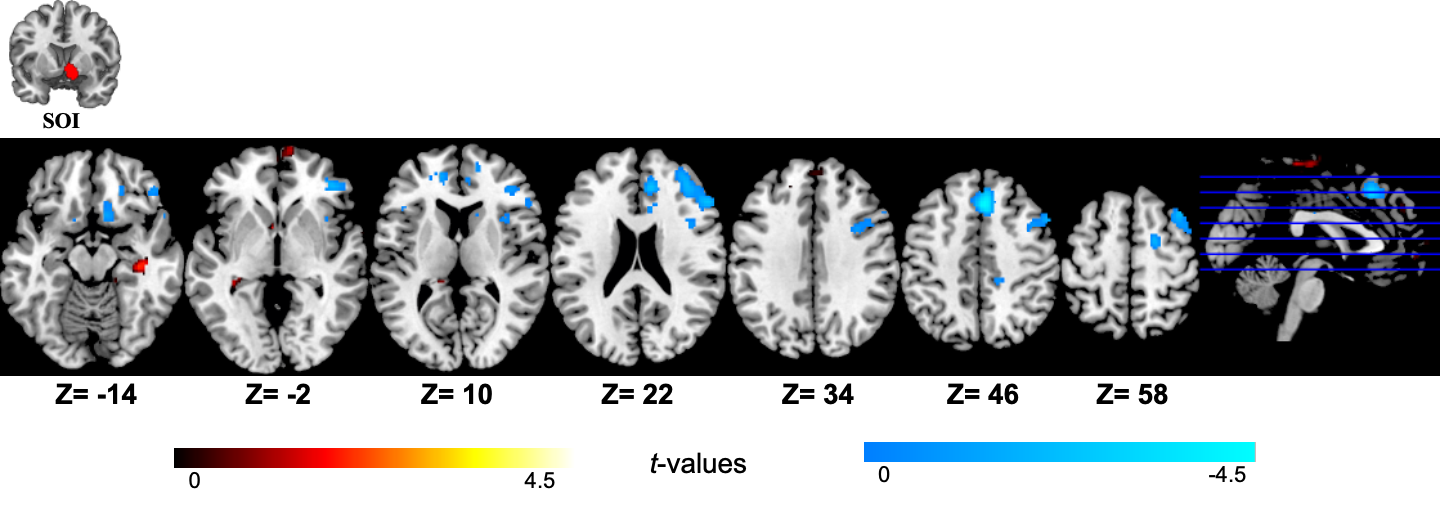


**Supplemental Figure 3.** Group Differences in Resting-state Functional Connectivity with NAcc Seeds of Interest. IP demonstrated less right NAcc-medial MFG/supplemental motor, right NAcc-right OFC, and right NAcc-right lateral superior MFG connectivity compared to HC (in blue/teal). There were no significant group differences in left NAcc connectivity.

**
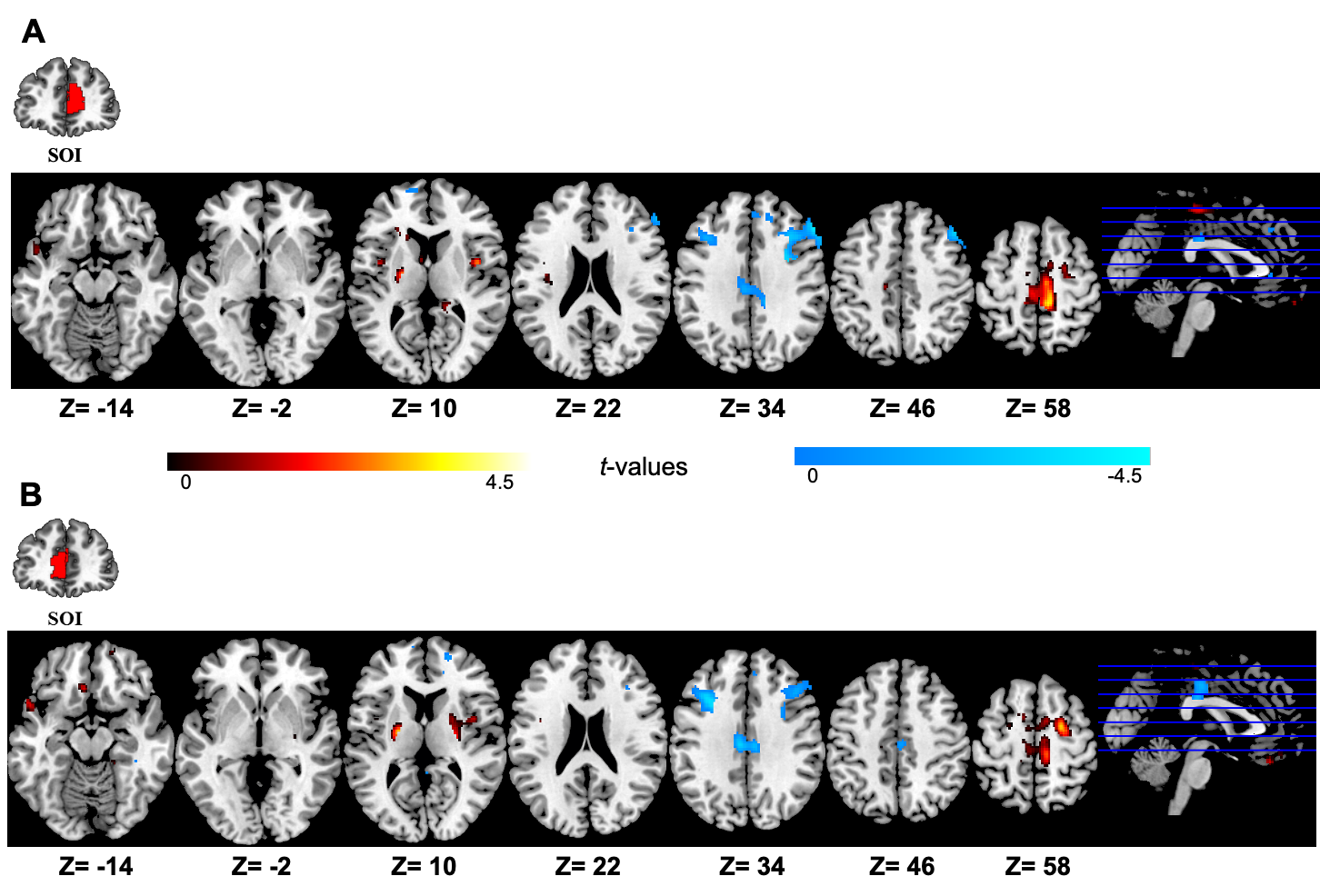
**

**Supplemental Figure 4.** Group Differences in Resting-state Functional Connectivity with ACC Seeds of Interest. Panel A shows that IP demonstrated greater right ACC-left superior temporal gyrus and right ACC-bilateral paracentral gyrus connectivity compared to HC (in red/yellow), while IP demonstrated less right ACC-MFG and right ACC-lateral MFG connectivity compared to HC (in blue/teal). Panel B shows that IP demonstrated less left ACC-MFG and left ACC-MCC connectivity compared to HC (in blue/teal).
